# Piezo1: opening the way to preventing muscle atrophy

**DOI:** 10.1172/JCI159668

**Published:** 2022-05-16

**Authors:** Ravi Jagasia, Kathryn R. Wagner

**Affiliations:** 1 Pharma Research and Early Development and; 2 Product Development Neuroscience, F. Hoffmann-La Roche, Basel, Switzerland.

## Abstract

The loss of skeletal muscle mass and size, or muscle atrophy, is a common human experience, linked to disability, for which there are no widely accepted pharmacological therapies. Piezo1 is a mechanosensitive cation channel that opens upon alteration of the plasma membrane lipid bilayer, such as through increased membrane tension. In this issue of the *JCI*, Hirata et al. identified Piezo1 and its downstream effectors, Krüppel-like factor 15 (KLF15) and interleukin-6 (IL-6), as an important signaling pathway in a murine model of disuse atrophy. Through genetic and pharmacological modulation of the pathway, the authors demonstrated that immobilization resulted in downregulation of Piezo1 and basal intracellular calcium concentration ([Ca^2+^]_i_), increasing expression of *Klf15* and its downstream target *Il6* and thereby inducing muscle atrophy. Piezo1 has been considered a therapeutic target for diverse disorders, including atherosclerosis and kidney fibrosis, and with this publication should now also be considered a viable target for disuse atrophy.

## Muscle atrophy

Skeletal muscle atrophy triggers frailty, disability, and death across the lifespan and across the globe. It is associated with numerous etiologies, including chronic systemic disease, disuse, aging, denervation, and intrinsic disorders of muscle, thus affecting a large proportion of humanity. Despite the widespread prevalence and considerable consequences of muscle atrophy in terms of quality and quantity of life, there are very few therapeutic options beyond rehabilitative and nutritional therapies (1). Muscle atrophy, while not homogeneous in its etiology or pathophysiology, is recognized to reflect predominantly a shift in balance between protein synthesis and degradation, principally driven by the interaction of the anabolic insulin-like growth factor-1 (IGF-1)/protein kinase B (Akt)/mammalian target of rapamycin (mTOR) pathway and the catabolic transcription factor forkhead box O (FoxO) and atrogenes (such as MuRF1 and MAFbx; refs. 2, 3). Other important modulators of muscle mass, many of which interact with the IGF-1/Akt/mTOR pathway, include myostatin, androgens, AMPK/PGC1α, IKKβ/NF-κB, and inflammatory cytokines (4–9).

**Figure 1:**
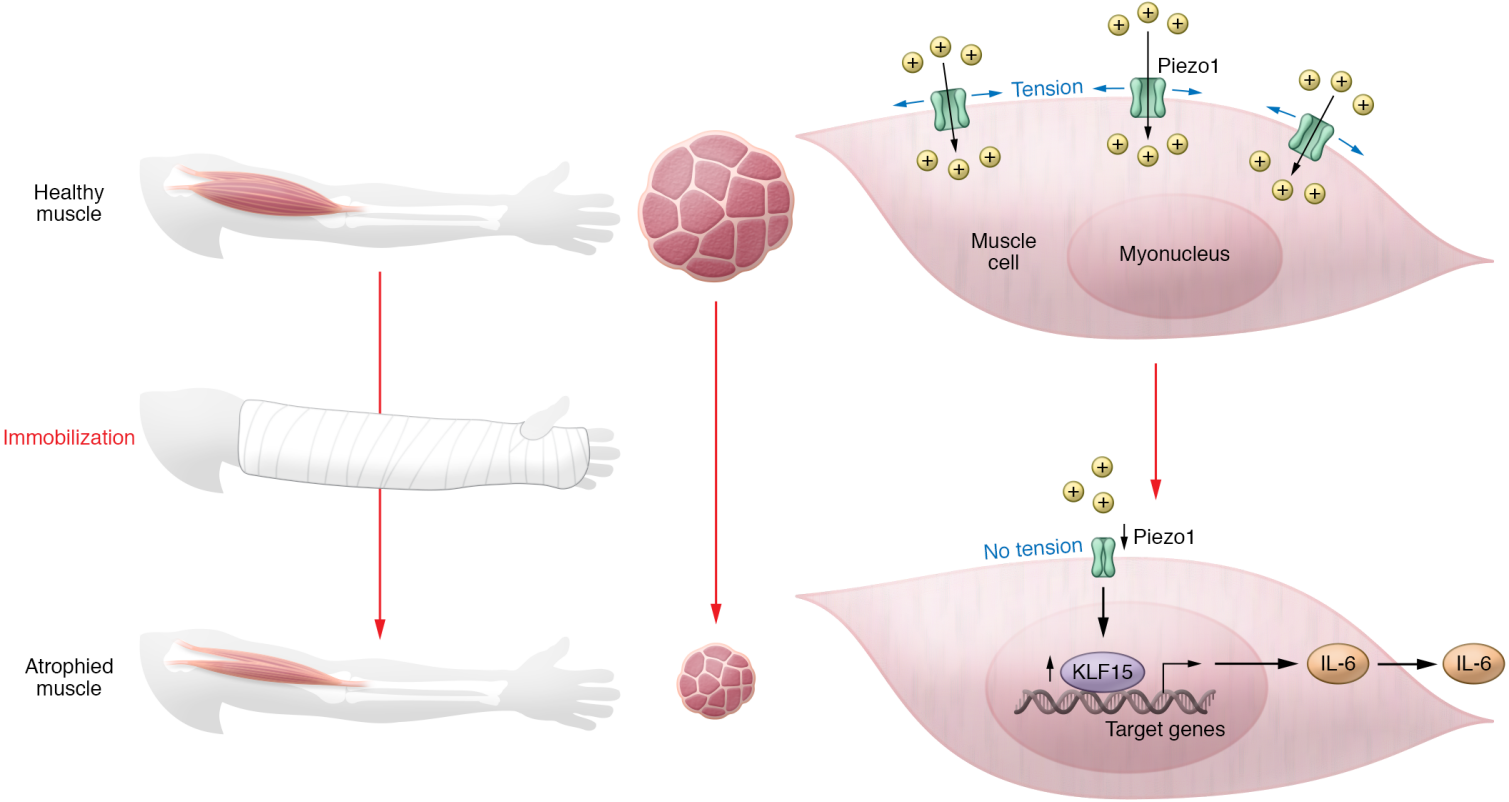
A reduction in mechanical stimulation during immobilization leads to downregulation of Piezo1 and muscle atrophy. During muscle immobilization, such as through limb casting, the muscle atrophies primarily through a reduction in myofiber size. Hirata et al. (10) provide evidence that muscle atrophy occurs through decreased expression and activation of the cation channel Piezo1, which is sensitive to mechanical tension. Absent Piezo1 activation, an increase in the transcription factor KLF15 modulates the expression of multiple target genes, including *IL6*.

In this issue of the *JCI*, Hirata et al. profile an emerging, and potentially druggable, signaling pathway regulating muscle atrophy. The authors performed a series of comprehensive genetic and pharmacological experiments identifying the Piezo1/KLF15/IL-6 pathway mediating muscle atrophy following immobilization, such as occurs with limb casting (10). While the transcription factor Krüppel-like factor 15 (KLF15) and the cytokine interleukin-6 (IL-6) have been independently implicated in some forms of muscle atrophy, the association of Piezo1 with muscle atrophy represents an upstream event (11, 12). Piezo1 is a mechanosensitive cation channel that opens upon alteration of the plasma membrane lipid bilayer, such as through increased membrane tension (13). Hirata et al. proposed a process by which a reduction in mechanical stimulation during immobilization leads to downregulation of Piezo1. Reduced Piezo1 channel activity and gene expression would lower basal intracellular calcium concentrations ([Ca^2+^]_i_), increase *KLF15* expression and, through KLF15 binding to the promoter region of *IL6*, increase IL-6–induced muscle atrophy (Figure 1). This narrative was supported by a dramatic reduction in *Piezo1* and an increase in *Klf15* and *Il6* mRNA in skeletal muscle after limb immobilization in mice. The authors demonstrated that Piezo1 in myotubes was activated by mechanical stimuli and involved in the maintenance of basal [Ca^2+^]_i_. GsMTx-4, a pharmacological inhibitor of Piezo1, phenocopied atrophy induced by increased expression of *Klf15* and *Il6* while conversely, Yoda-1, an allosteric positive modulator of Piezo1, blunted the upregulation of the same genes after immobilization in mice. Downstream, tissue-specific knockout of *Klf15* abrogated *Il6* upregulation and muscle atrophy. Neutralizing antibodies against IL-6 prevented immobilization-induced upregulation of atrogenes and muscle atrophy. To address the translatability of these preclinical findings, human muscle biopsy samples from patients casted for fracture were compared with those from patients several months out from fracture and casting and the authors demonstrated that *PIEZO1* mRNA was reduced, *KLF15* showed a trend toward increased expression, and *IL6* and various atrogenes were increased (10).

During immobilization, downregulation of *Piezo1* and upregulation of *Klf15* were observed in the non–satellite cell fraction, which contained multinucleated myofibers, fibroblasts, and endothelial cells. Conversely, *Piezo1* and *KLF15* changes were not observed in the satellite cell fraction, which corresponded with *Pax7*^pos^ muscle stem cells (MuSCs), suggesting that the Piezo1/KLF15/IL-6 pathway of muscle atrophy occurs predominantly if not exclusively in myofibers (10). However, recently, two intriguing papers have also demonstrated key roles of Piezo1 in MuSC function, implicating Piezo1 in MuSC fusion and muscle regeneration (14, 15). MuSCs play a major role in muscle growth and regeneration but there is little evidence to support their role in acute muscle atrophy. Therefore, currently, from the flurry of recent papers on Piezo1 in skeletal muscle, it appears that the cation channel is critical in both myofiber and MuSC physiology where it is similarly sensitive to stretch and responsible for calcium influx but where it potentially activates different downstream signaling, gene expression, and cell functions.

## A druggable target for muscle wasting

Piezo1 represents a potential druggable target in the quest to halt muscle wasting. There are still mechanistic gaps in the understanding of how Piezo channels are modulated by muscle activity. However, since Piezo1 is linked to multiple diseases, there is already good knowledge on the druggability of the channel with a wealth of structural and functional data, including an understanding of potential allosteric sites that can support rational design of putative isoform-selective Piezo modulators (16). Until then, the safety profile of Piezo1 modulators remains to be determined, since it is rather promiscuously expressed and since there are data suggesting that modulation of both Piezo1 and KLF15 may need careful titration to avoid adverse effects on muscle growth and regeneration (15, 17). Notwithstanding the fact that there are certainly many downstream effectors of Piezo1 activity, IL-6 may be a good alternative target in this pathway and there are already several approved IL-6 inhibitors, including anti–IL-6 receptor and anti–IL-6 monoclonal antibodies (18).

Hirata et al. identify Piezo1 as a relevant upstream target in muscle atrophy, warranting future exploration (10). Like any good study, it raises many additional questions: (a) How is Piezo1 modulated in mature myofibers and satellite cells in atrophy, degeneration, and regeneration? (b) Is there crosstalk between this pathway and other major known pathways directing muscle atrophy? (c) How does Ca^2+^ influx from the Piezo1 channel modulate *KLF15* expression and what is the interplay with other Ca^2+^ sources, such as through voltage-gated calcium channels and the ryanodine receptor? (d) Is this pathway important in both acute and chronic processes involved in muscle atrophy? (e) And perhaps most importantly, are these findings generalizable to other etiologies of muscle atrophy? Hopefully, many of these questions will be answered while moving molecules on the path to clinical development.

## Version 1 05/16/2022

Electronic publication
